# Reflexes from Lower Molars

**Published:** 1899-10

**Authors:** James Truman

**Affiliations:** Philadelphia


### REFLEXES FROM LOWER MOLARS.[1]

BY JAMES TRUMAN, D.D.S., PHILADELPHIA.

THE causes of reflex disturbances have been sufficiently studied as a collateral branch of dental pathology, and it may seem a useless expenditure of the time of this organization to attempt to add anything to the literature of the subject.

When the student of dental lesions comes to investigate the various books that are supposed to treat upon the reflexes of the teeth, he finds himself narrowed down to a small compass. He may critically examine all the works on dental pathology and oral surgery in the English language, issued in recent years, to be surprised with the fact that where this subject received extended notice it has been mainly confined to infantile dentition, or it has been regarded as of secondary importance, and by some it is held to be unworthy of pathological consideration.

The eruption of the permanent molars, aside from their histological interest, have secured but slight attention. It is true that the consideration of the intimate connection between the lesions occurring in teeth and the neuroses of the fifth nerve have, of recent years, been more thoroughly studied, but it has been more from the histological side, hence abnormal formations in the pulp have received a full degree of attention from dental writers, as these enter into daily practice. The exceptional conditions are, however, passed by as of minor importance, or they have been entirely overlooked.

It is somewhat strange that the lesions of dentition have been treated by the majority of medical writers with slight consideration. The opposition by the medical profession to the generally accepted position of the dental, that the reflex disturbance of dentition is of vital importance, can be understood, for medicine, until very recently, has entirely underrated, if it has not entirely ignored, the

---

[1] Read before the National Dental Association, held at Niagara Falls, August 1 to 4, 1899.

oral cavity as a source of disease. This should not, perhaps, be regarded as remarkable, since the origin of disease in general has only been studied scientifically during the past twenty-five years, and is even now in its infancy. Medicine has been based on symptomatology, the effect, and not the cause, receiving attention. It is, therefore, not a special source of wonder that the neuralgias, the tissue-disturbers, the new foci of inflammation, having their origin in the teeth, have received only a moderate degree of thought.

While this is true of medicine in general, the specialist, the stomatologist, must share the responsibility of neglect.

The views that this paper proposes to consider are, in the main, written to enforce the idea that more attention should be given to the eruption of the molars than has been accorded them. The same laws of growth are manifested here as in the first dentition, and are equally responsible for serious disturbance along the course of nerve-connections. Consideration will alone be given to the lower molars, for the question of growth does not apply with equal force to the upper set. It is, however, recognized that nerve irritation proceeds from these as it does from all the teeth disturbed from their normal relations.

The process of growth, whether in vegetable or animal tissues, is one of the most interesting in nature. John Tomes ("Dental Physiology and Surgery," 1848), in writing of this force, says, "It would be difficult to estimate the actual amount of mechanical force developed by a growing tooth, but we may form some estimate by observing the force generated by a growing vegetable." If this force of growth be applied to the teeth, and it must be so applied, it can be well understood why, in dentition, reflex disturbance occurs. The whole question of treatment might be condensed into one word, —*room.* With space sufficient for the development of teeth all dentition would simply be a physiological and not a pathological process. It is, therefore, a question of space or room for development.

Growth proceeds from the first layer of enamel and dentine to the last of cementum, and in proportion as the enlargement proceeds must the tooth be given space for its formation, and, to accomplish this, the gumward advance must be regular and with no opposing obstacles. In deciduous dentition the obstacles are confined to the gum tissue, and these teeth are equally with the permanent a source of reflex disturbance.

The student of these phenomena has, therefore, a comparatively simple problem to solve. His duty is first to study the anatomy of the jaw, and the process of growth in the jaw primarily, and to follow this by that of the teeth. He cannot omit in this consideration the position of development and the resulting changes that take place in its progress.

The development of the first permanent molar takes place about the tenth week of fœtal life, and from this period until the sixth year subsequent to birth is gradually formed into the largest molar of the series. The broad occlusal surface of this tooth would seem to indicate that at the period of eruption it would be a special subject for pathological disturbance; but it is well known to be more generally free from reflexes than any other of the developing permanent teeth in the inferior maxilla. If the jaw has been examined, it will be found that this exemption is clearly explainable by the width of the jaw, measuring from the gum tissue to the inferior dental canal. The only obstruction to receive serious consideration is the tense overlying gum. This, while resistant, gradually yields to pressure, resorption taking place, and while it may retard eruption, it cannot, as a general rule, cause pressure upon the exposed pulp or upon the inferior dental nerve. Hence there is a marked and almost uniform freedom from neuroses in this tooth, and it may be passed as an unimportant factor in pathological disturbance.

The second, or, as it is commonly misnamed, the twelfth-year molar, has an entirely different place in development, and in consequence more fully exemplifies the importance of room for growth than any of the series, not even excepting the third molar, which is usually regarded as the prime factor in neuralgic lesions. From an extended examination of the literature of the subject, nowhere have I found this tooth noticed as a cause of reflex conditions. It is, therefore, worthy of study in its several relations, origin, development, and eruption.

The growth of the jaw posteriorly carries the germs of these teeth well up in the rami, and as development proceeds both in jaw and teeth there is a gradual descent towards the angle of the jaw. The period of passing this point, through development of the latter, to assume the direct vertical position is the critical period. At this time the crowns are fully formed externally and the roots have developed to half their normal length. If the growth of the jaw is in harmonious relation to the development of the teeth, this period may be passed without notice; but this is not always the case. The growth of the roots may have increased more rapidly than that of the jaw, and in passing the angle they may, and frequently do, impinge upon the inferior dental nerve, or the exposed pulps may possibly be forced by the exceedingly tense gum against the bone envelope, in either case resulting in serious reflex suffering to the individual. The passage of this critical point by these teeth occurs about the ninth year.

The characteristic phenomena at this time are marked cerebral disturbance, resulting in convulsions, epilepsy, chorea, insanity, hysteria, otalgia, ocular diseases, and a long list of more or less aggravated lesions. In the before-mentioned work of Tomes, the author gives this as his opinion on reflex manifestations: "Before serious and painful results can arise there must be a concurrence of many circumstances. The patient must be of a strumous constitution or predisposed to inflammatory action, or the nervous system must be peculiarly susceptible to excitement. In the latter case epileptic fits may supervene. . . . That epilepsy does arise in some cases from this cause is proved by its appearing when the teeth are passing towards the surface and disappearing when passage is given to the teeth."

Dewees ("Physical and Medical Treatment of Children") makes this admission: "And though the teeth cut [lanced] upon may yet be *remote from the surface*, still the operation may be of the greatest possible advantage by dividing the membrane, . . . and the disturbance of the system is quieted from the moment the gum is divided." That the phenomena have been attributed to other causes is not surprising, in view of the fact that comparatively slight attention has been given to dentition as a whole and the permanent teeth in particular.

That this statement is not based on mere conjecture, attention is called to the anatomical relations of the second molar with the jaw. A very cursory examination will bring conviction as to the truth of the conclusions at which I have arrived. My attention was originally called to this tooth as a prominent cause of reflexes at this age by several cases coming under my care, which led to a more critical examination to discover, if possible, the cause and the remedy.

It is a well-understood fact by the more advanced dental observers that the nervous disturbances at this age, about nine years, have generally their origin in the teeth, and so certain are these as to the cause that the intelligent practitioner never fails to examine for obstructed dentition when called to diagnose a case. Dr. C. N.

Peirce mentions a case, a child of a distinguished physician, and afflicted with chorea. The cause was diagnosticated as a retained deciduous molar. The father was not able to comprehend that this retention could cause the nervous symptoms in the child. The extraction, however, demonstrated the diagnosis to be correct, and all reflex phenomena ceased.

In the case of the second molar, as well as others similarly situated, the relief must come by affording room for growth.

It is apparent that this is a difficult procedure with this tooth. It is deeply embedded in the surrounding tissues, and it would seem impossible to afford any immediate relief. The first case that presented and which claimed serious consideration was a member of my own family. At the ninth year this child was, without apparent cause, affected with violent convulsions that assumed an epileptic character. The seizures were prolonged, but days frequently intervened between them. The study given the case developed the hypothesis that the second molar was the pronounced factor in the cerebral disturbance. To verify this theory the child was carefully watched, and upon indications of an approaching nervous storm the lance was deeply inserted over the position of this molar. The result was entirely confirmatory of the diagnosis, and by care in watching symptoms and a repetition of the lancing, whenever necessary, the child passed the critical period without further reflex phenomena. Since that time other cases have been under my care, and the same simple method of treatment has been followed with equally good results. During the present spring a child of nine years was brought to me, suffering intensely with pain in both ears. Examination of the teeth failed to show any cause for this, in exposed pulps or other lesions. These having been excluded from the possibility of causing the suffering, it was decided that the origin of the pain was reflex and must be from pressure upon the pulp of the partially developed second molars, or impingement of both upon the inferior dental nerve. The lance was at once applied to both right and left, with immediate relief, and without any subsequent recurrence of pain now several months since the operation was performed.

There is, therefore, no question in my own mind but that dentists are called upon to watch carefully the development of the second molar in childhood, and advise, the only remedy, free lancing.

The books are full of the sage advice that lancing should not be performed until the tooth manifests itself by forcing the gum above it into a rounded prominence. Lancing is indicated at *all stages* of development, whether upon the deciduous or permanent teeth, and should be resorted to as frequently as the symptoms indicate reflex phenomena. Dr. Dewees (quoted previously) recognized this, and he is the only medical writer with whom I am familiar who has comprehended this important point.

The third molar is so frequently the cause of reflexes that it would seem almost a useless expenditure of time to reiterate a well-known fact, and yet an examination of all the books on dental pathology and oral surgery reveal a great deficiency in the treatment of this subject. While this is true, I must refrain from its extended consideration before this intelligent body. Suffice it to say that these teeth, like the second molars, find their place of origin in the rami of the inferior maxillæ, and have difficulty in assuming the direct vertical position. The two principal positions assumed by this tooth, causing general nervous irritation, are the oblique and the horizontal. The former is not a frequent cause of reflexes, and while it may produce these by impingement upon the inferior dental nerve, it more often effects this by producing unobserved decay at the cervical border of the second molar, and eventually pressing upon the pulp. The difficulty of diagnosticating this must be evident, for as decay proceeds the tooth advances, filling completely the cavity and causing the lesion to be overlooked, until neuralgic pains indicate some cause requiring the attention of the dental surgeon.

The most serious of all the malpositions of this tooth is the horizontal. The tooth in passing down from the ramus strikes the posterior root of the second molar and there must stop. Deeply embedded in the jaw, with the roots closely impinging on the inferior dental nerve, there is but one possibility, that of pressure upon that nerve, and that continually and more forcibly as growth proceeds. The reflex lesions are, as you are well aware, of the most serious character. The important matter to consider here is the diagnosis.

It is quite evident that a tooth thus situated produces its disturbing effect through lack of room, and that this room cannot be secured as long as all the teeth on that side are in place. If, then, the entire series, from the second molar to the canine, are in position at the age of eighteen to twenty-four, with lancinating and intermittent pains extending throughout the fifth pair, and with no visible signs of a developing tooth, it is evident that the third molar is abnormally developed in the position named. An explorer passed down posterior to the second molar will settle this fact. If, however, the second molar be absent, or it is in place and the first molar absent, it remains reasonable that the third molar is not the cause of the neuralgia. The force of growth, as before stated, is quite sufficient to change the second molar from its normal position, and thus give room for the development of the third molar. If, however, both first and second molars are in place, the inference may be drawn that the third molar will not have sufficient force to move two such powerfully implanted teeth far enough to afford relief.

The importance of this malposition cannot be over-estimated. Forget mentions a number of serious cases, and almost every practitioner of dentistry has seen cases of obscure reflex pain, which may not have been fully comprehended. There is no lesion more easily treated than this, and yet it is one the general surgeon fails almost universally to diagnosticate correctly. A case which I reported many years ago may be worth repetition here as an illustration of this statement. A patient was sent to me for examination for a most obstinate case of neuralgia, which had resisted medical and surgical care for twenty years. The physician who sent her expressed himself as hopeless of having her relieved. The woman was a physical and almost a mental wreck. Feeble and emaciated, vision very defective, hearing with difficulty, she presented a most dejected and hopeless appearance. The examination of the mouth gave no indication of a third molar. All the other teeth were in place. An explorer was passed down posterior to the second molar, and the third was found occupying a horizontal position, abutting against the roots of the second molar. The patient was informed that the only remedy was the extraction of the second molar. No opposition was made to this, and the tooth was at once removed. The effect was immediate, all pain ceasing, and in twenty-four hours the third molar had advanced and filled up the socket of the second molar. The patient in a month's time had recovered her hearing and the eyesight was greatly improved, and her condition physically and mentally had become normal, pain having ceased entirely. She should have been saved this twenty years of torture.

At the time this case was under care the X-rays were an unknown factor in diagnosis. It is unnecessary to state here that this, at present, would have decided such a malposition with unerring certainty. This paper is not written for the favorably situated in large centres of population, where this aid can readily be obtained, but for those in isolated practice, who will need to decide cases, as they occur, by the older and, perhaps, obsolete methods.

The reflexes from dentition, exposed pulps, calcification in pulps, and from other causes, have received due attention from various writers, but those interested will find very full reports of cases given by Brubaker, "American System of Dentistry," Arkövy, "Diagnostik der Zahnkrankheiten," in appendix prepared by Dr. Georg Creniceneau, and in Wedl's "Pathology of the Teeth." It is, therefore, not deemed necessary to do more than allude to the fact that the recorded cases are so numerous that he who fails to recognize their importance is not fully grounded in the pathological possibilities contingent upon the abnormal changes in teeth. The subject is still obscured by imperfect observations, but sufficient is known to afford a basis for intelligent practice upon the general subject.

The reflex disturbances occasioned by the various recognized lesions have not been considered, as these are well understood and require no further elucidation. The main purpose of this paper has been to direct thought upon this subject, and more especially to two facts, first, that relief should not be delayed until the teeth manifest themselves at the gum border, but in all cases of disturbed dentition, whether it be with deciduous or permanent teeth, the lance should be freely used, and that at the first symptoms of reflex disturbance; and, secondly, to call attention to the wholly neglected second molar as a cause of many of the serious reflexes about the ninth year.

